# The eyes see what the mind seeks: a systematic review of abdominal imaging findings in patients with COVID-19

**DOI:** 10.1259/bjr.20201220

**Published:** 2021-07-14

**Authors:** Lokesh Agarwal, Ayushi Agarwal, Shailesh Advani, Varidh Katiyar, Aprajita Chaturvedi, Kumble Seetharama Madhusudhan

**Affiliations:** 1 Department of Gastrointestinal Surgery and Liver Transplantation, All India Institute of Medical Sciences (AIIMS), New Delhi, India; 2 Department of Radiodiagnosis and Interventional Radiology, All India Institute of Medical Sciences (AIIMS), New Delhi, India; 3 Department of Oncology, Lombardi Comprehensive cancer Center, Georgetown University, Washington, DC, Washington, DC, USA; 4 Department of Neurosurgery, All India Institute of Medical Sciences (AIIMS), New Delhi, India; 5 Department of Surgery, All India Institute of Medical Sciences (AIIMS), New Delhi, India

## Abstract

**Objectives:**

With the increasing recognition of gastrointestinal (GI) manifestation of coronavirus disease-19 (COVID-19), various abdominal imaging findings are increasingly being noted. We scoped the existing literature on the abdominal imaging findings in COVID-19.

**Methods:**

A systematic literature search was performed on PubMed, Embase, Google scholar and World Health Organization COVID-19 database.

**Results:**

35 studies were included in the final descriptive synthesis. Among the studies reporting positive abdominal imaging findings in patients with COVID-19, majority described imaging abnormalities of the GI tract (16 studies), of which bowel wall thickening was most frequently reported. Other findings noted were abdominal imaging manifestations of bowel ischemia with thrombosis of the splanchnic vasculature, and imaging features suggestive of pancreatitis. Imaging findings suggestive of solid organ infarction were reported in nine studies. An association between imaging evidence of hepatic steatosis and COVID-19 was noted in three studies. Incidental lung base findings on abdominal imaging were noted in 18 studies, where patients presented with predominant GI symptoms. The most common finding was bilateral ground glass opacities (90.7%) with predominant multilobar (91.1%) and peripheral (64.4%) distribution.

**Conclusion:**

This systematic review provides insight into the abdominal imaging findings in patients with COVID-19. Knowledge of these imaging manifestations will not only help in further research but also will aid in curtailing transmission of the SARS-CoV-2. Further prospective studies are needed to gain better insight into the pathophysiology of these imaging manifestations.

**Advances in knowledge:**

This review highlights the abdominal imaging findings in patients with COVID-19, to gain insight into the disease pathophysiology and gear the abdominal radiologist through the pandemic.

## Introduction

The world has faced a great challenge in public health with the Coronavirus Disease-19 (COVID-19) pandemic. Severe acute respiratory syndrome coronavirus-2 (SARS-CoV-2) is known to infect the respiratory epithelium and cause respiratory illness ranging from mild flu to severe interstitial pneumonia and acute lung injury. The virus targets the type II alveolar cells abundantly expressing the angiotensin converting enzyme II (ACE-II) receptors.^1^ Gastrointestinal (GI) involvement was first reported by Xiao et al^2^ where they demonstrated viral ribonucleic acid in the stool specimen of patients with COVID-19. Further studies showed that GI tract may be a target organ of SARS-CoV-2 as the ACE-II receptors are abundantly expressed on the epithelium of both small and large intestine.^2,3^ Despite the increasing recognition of the abdominal manifestations, the literature on abdominal imaging in COVID-19 is sparse and scattered. The abdominal or emergency radiologist may be the first to suspect and suggest a diagnosis of COVID-19 in a patient presenting with atypical symptoms. Knowledge of the abdominal imaging findings of patients infected with SARS-CoV-2 and presenting with GI symptoms may help in understanding the pathophysiology of the disease, and thus aid in better prognostication of this subset of patients. This review was conducted with the aim to scope the existing literature on the abdominal imaging findings in confirmed COVID-19 cases. Prior to the start of this review, we checked to make sure that there was no existing or ongoing review on this topic.

## Methods and materials

This systematic review was conducted in accordance with the Preferred Reporting Items for Systematic Reviews and Meta-Analyses (PRISMA) guidelines.^4^ The review was registered on PROSPERO (CRD42020193885). An initially planned meta-analysis could not be performed because of the inadequacy of published literature for quantitative synthesis of data.

### Data source and literature search

Literature databases including PubMed (Medline), Embase (Ovid) and selected parts of the Cochrane library relating to COVID-19 were searched from inception of COVID-19 to August 2, 2020. An updated literature search was performed on September 5, 2020. Keywords that made up our search included (“coronavirus” OR “nCoV” OR “2019-nCoV” OR “COVID-19”) AND (“abdominal imaging” OR “abdominal CT” OR “abdominal ultrasound “OR “abdominal MRI”). The detailed search strategy is mentioned in the Supplementary Material 1, supplementary appendix. Furthermore, COVID-19 publications in the WHO publication database, *The Lancet* COVID-19 Resource Centre*, JAMA, BMJ* were screened for relevant publications. Additional articles were retrieved by screening the reference lists of the included studies and reviews. We hand-searched conference and meeting abstract lists and the relevant articles were cross-referenced to identify relevant studies. There was no language restriction in the searches. There were no restrictions in terms of country or publication date.

Supplementary Material 1.

### Research question

The following research questions guided this systematic review: What are the abdominal imaging findings in patients with COVID-19 infection? Do the abdominal imaging findings correlate with the severity of COVID-19 infection?

### Eligibility criteria

All studies included in this systematic review were peer-reviewed, and included original research, case series and case reports, that described or provided an overview of abdominal imaging findings among confirmed COVID-19 cases. Only the articles which described abdominal imaging findings in confirmed COVID-19 cases were included in the review. No age-related exclusions were made.

### Study selection

Study selection was performed by two independent investigators (LA, AA). In the final review, the studies or articles which comprised of patients with confirmed COVID-19 and had reported abdominal imaging (ultrasound, CT, or MRI) findings were included. Discrepancies between reviewers were resolved through consensus with a third reviewer (KSM).

### Data extraction and synthesis

The following data categories were collected when available: study design, country, patient demographics, clinical presentation, imaging modality and findings on abdominal imaging, severity grading of COVID-19 infection (Supplementary Table 1). One of the reviewers performed the data extraction (AA) and the other reviewer assessed the accuracy of the extracted data (AC).

Supplementary Table 1.

### Risk of bias

Two reviewers (SA, VK) independently rated the quality of the included studies using the National Institutes of Health Quality Assessment Tools^5^ (Tables 1–3). Any difference was resolved by consensus, in the presence of the third reviewer (KSM). Studies rated as poor quality on the NIH quality assessment tool were excluded from the final qualitative analysis.

**Table 1.** Quality assessment of the included observational cohort studies based on the National Institutes of Health (NIH) quality assessment tool

First author (Reference no.)	Question (NIH assessment tool)^a^														Overall rating	
	1	2	3	4	5	6	7	8	9	10	11	12	13	14	Reviewer#1	Reviewer#2
Goldberg-Stein et al^6^	Yes	Yes	Yes	NA	No	Yes	No	No	No	No	Yes	No	No	No	Fair	Fair
King et al^7^	Yes	Yes	Yes	NA	No	Yes	No	No	No	No	Yes	No	No	No	Fair	Fair
Shea et al^8^	Yes	Yes	Yes	NA	No	Yes	No	No	No	No	Yes	No	No	No	Fair	Good
Norsa et al^9^	Yes	Yes	Yes	No	No	Yes	No	No	No	No	Yes	No	No	No	Fair	Fair
Bhayana et al^10^	Yes	Yes	Yes	Yes	No	Yes	No	No	No	No	Yes	No	No	No	Good	Fair
Palomar-Lever et al^11^	Yes	Yes	Yes	No	No	Yes	No	No	No	No	Yes	No	No	No	Good	Fair
Uchida et al^12^	Yes	Yes	Yes	No	No	Yes	No	No	No	No	Yes	No	No	No	Fair	Fair
Dane et al^13^	Yes	Yes	Yes	CD	No	Yes	CD	CD	CD	No	Yes	CD	CD	No	Fair	Good
Shiralkar et al^14^	Yes	Yes	Yes	No	No	Yes	No	No	No	No	Yes	No	No	No	Fair	Fair
Xiao et al.^15^	Yes	Yes	Yes	NA	No	Yes	No	No	No	No	Yes	No	No	No	Fair	Fair
Sellevol et al.^16^	Yes	No	Yes	CD	No	No	CD	CD	CD	No	No	CD	CD	No	Fair	Fair
Liu et al.^17^	Yes	Yes	Yes	Yes	No	Yes	No	No	No	No	Yes	No	No	No	Fair	Good

CD, Cannot determine; NA, Not applicable; NIH, National Institutes of Health; NR, Not reported.

Source: National Heart, Lung, and Blood Institute; National Institutes of Health; U.S. Department of Health and Human Services).

a The NIH Quality Assessment Tool for Observational Cohort and Cross-Sectional Studies^5^ includes 14 questions: 1 = Was the research question or objective in this paper clearly stated? 2 = Was the study population clearly specified and defined? 3 = Was the participation rate of eligible persons at least 50%? 4 = Were all the subjects selected or recruited from the same or similar populations (including the same time period)? Were inclusion and exclusion criteria for being in the study prespecified and applied uniformly to all participants? 5 = Was a sample size justification, power description, or variance and effect estimates provided? 6 = For the analyses in this paper, were the exposure(s) of interest measured prior to the outcome(s) being measured? 7 = Was the timeframe sufficient so that one could reasonably expect to see an association between exposure and outcome if it existed? 8 = For exposures that can vary in amount or level, did the study examine different levels of the exposure as related to the outcome (*e.g.,* categories of exposure, or exposure measured as continuous variable)? 9 = Were the exposure measures (independent variables) clearly defined, valid, reliable, and implemented consistently across all study participants? 10 = Was the exposure(s) assessed more than once over time? 11 = Were the outcome measures (dependent variables) clearly defined, valid, reliable, and implemented consistently across all study participants? 12 = Were the outcome assessors blinded to the exposure status of participants? 13 = Was loss to follow-up after baseline 20% or less? 14 = Were key potential confounding variables measured and adjusted statistically for their impact on the relationship between exposure(s) and outcome(s)?

**Table 2.** Quality assessment of the included case–control studies based on the National Institutes of Health (NIH) quality assessment tool

First author (Reference no.)	Question (NIH assessment tool)^*a*^												Overall rating	
	1	2	3	4	5	6	7	8	9	10	11	12	Reviewer#1	Reviewer#2
Bari Dane et al^18^	Yes	Yes	No	CD	Yes	Yes	No	Yes	Yes	No	No	No	Fair	Fair
Medeiros et al^19^	Yes	Yes	No	No	Yes	Yes	No	Yes	Yes	No	No	No	Fair	Fair

CD, Cannot determine; NA, Not applicable; NIH, National Institutes of Health; NR, Not reported.

Source: National Heart, Lung, and Blood Institute; National Institutes of Health; U.S. Department of Health and Human Services).

a The NIH Quality Assessment Tool for Case–Control Studies^5^ includes 12 questions: 1 = Was the research question or objective in this paper clearly stated? 2 = Was the study population clearly specified and defined? 3 = Did the authors include a sample size justification? 4 = Were controls selected or recruited from the same or similar population that gave rise to the cases (including the same timeframe)? 5 = Were the definitions, inclusion and exclusion criteria, algorithms or processes used to identify or select cases and controls valid, reliable, and implemented consistently across all study participants? 6 = Were the cases clearly defined and differentiated from controls? 7 = If less than 100 percent of eligible cases and/or controls were selected for the study, were the cases and/or controls randomly selected from those eligible? 8 = Was there use of concurrent controls? 9 = Were the investigators able to confirm that the exposure/risk occurred prior to the development of the condition or event that defined a participant as a case? 10 = Were the measures of exposure/risk clearly defined, valid, reliable, and implemented consistently (including the same time period) across all study participants? 11 = Were the assessors of exposure/risk blinded to the case or control status of participants? 12 = Were key potential confounding variables measured and adjusted statistically in the analyses? If matching was used, did the investigators account for matching during study analysis?

## Results

### Overview of the included studies

**Figure 1.**
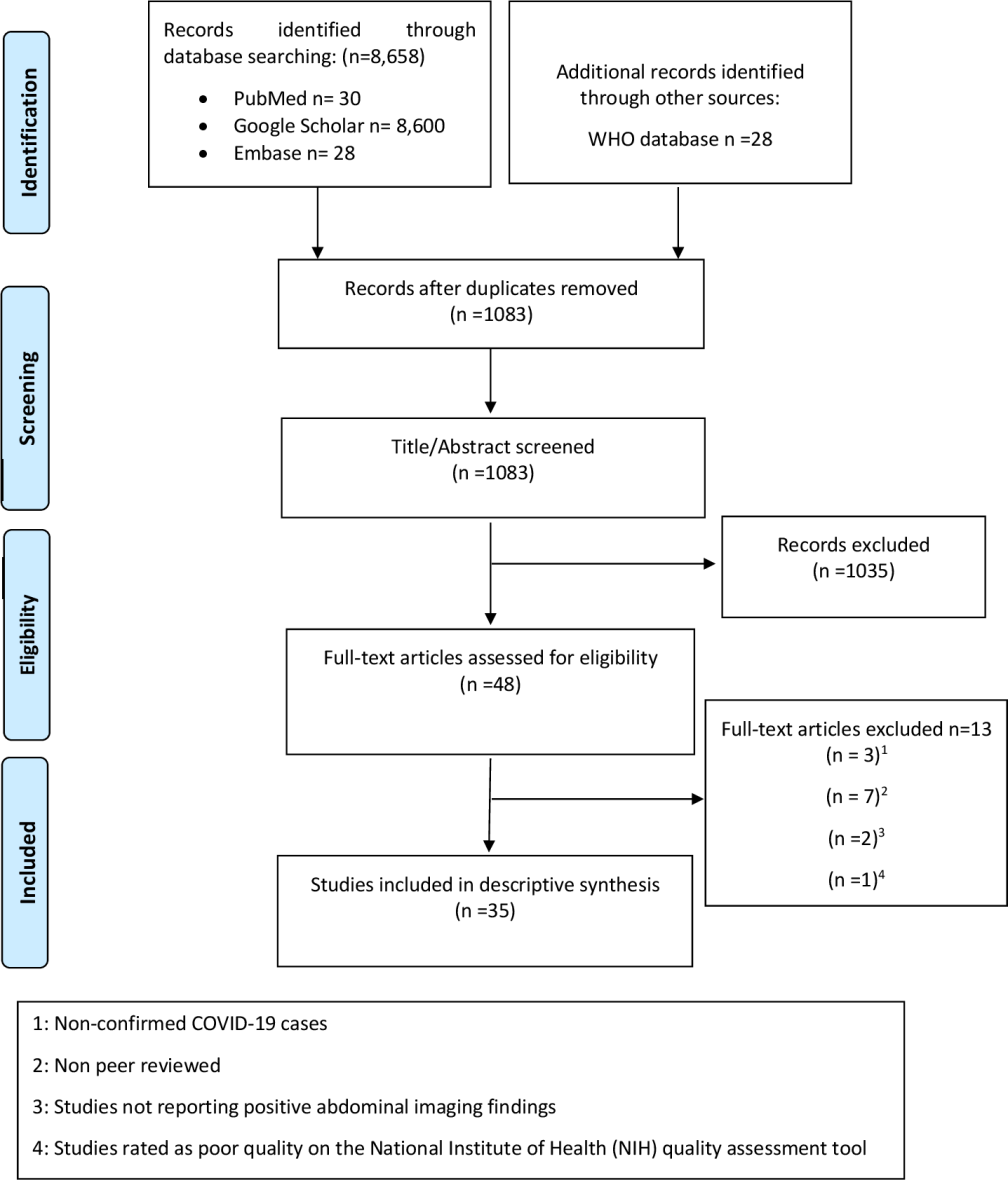
PRISMA flow diagram showing study selection process. PRISMA, Preferred reporting items for systematic reviews and meta-analyses; WHO, World Health Organization.

Only English language papers were identified. 35 studies consisting of 11 retrospective cohort studies (RCS), 2 retrospective case–control studies, 9 case series and 13 case reports, with a total of 801 COVID positive patients were included in the final review, as shown in PRISMA flow diagram (Figure 1). Characteristics of the included studies are presented in Supplementary Table 1. The number of the abdominal imaging modalities evaluated in the included studies was 721 CT scans.

### Abdominal imaging manifestations

A plethora of abdominal imaging findings in COVID-19 were reported in several studies (Table 4). These imaging manifestations have been grouped by the organ involved as shown below.

**Table 3.** Quality assessment of the included case series/reports studies based on the NIH quality assessment tool

First author (Reference no.)	Question (NIH assessment tool)a									Overall rating	
	1	2	3	4	5	6	7	8	9	Reviewer#1	Reviewer#2
Ahmed et al^20^	Yes	Yes	CD	CD	Yes	Yes	No	CD	Yes	Good	Good
Azouz et al^21^	No	Yes	NA	NA	Yes	No	CD	NA	Yes	Fair	Fair
Bessuti et al^22^	No	Yes	CD	CD	NA	Yes	NA	NA	Yes	Poor	Poor
Colino et al^23^	Yes	Yes	NA	NA	NA	No	CD	NA	No	Fair	Fair
Vu et al^24^	Yes	CD	CD	Yes	NA	NA	No	NA	Yes	Good	Good
Poggiali et al^25^	Yes	Yes	CD	Yes	Yes	Yes	CD	NA	Yes	Good	Good
Gahide et al^26^	Yes	No	CD	CD	No	Yes	No	NA	Yes	Fair	Fair
Ignat et al^27^	Yes	No	CD	No	NR	CD	CD	NA	Yes	Fair	Fair
Kim et al^28^	Yes	NA	NA	NA	CD	No	CD	NA	Yes	Fair	Fair
Mazrouei et al^29^	Yes	No	NA	NA	No	Yes	CD	NA	Yes	Fair	Fair
Pessoa et al^30^	Yes	Yes	CD	CD	NA	NA	NA	NA	Yes	Good	Good
Kumar et al^31^	Yes	NA	NA	NA	NA	Yes	NA	NA	Yes	Fair	Fair
Sattar et al^32^	Yes	Yes	CD	CD	Yes	Yes	CD	NA	Yes	Good	Good
Sendi et al^33^	Yes	No	NA	NA	No	Yes	CD	NA	Yes	Fair	Fair
Tay et al^34^	Yes	Yes	NA	NA	Yes	Yes	CD	NA	Yes	Good	Good
Siegel et al^35^	Yes	Yes	NA	Yes	Yes	Yes	CD	NA	Yes	Good	Good
Jaijakul et al^36^	Yes	Yes	NA	NA	Yes	Yes	No	NA	Yes	Good	Good
Voutsinas et al^37^	Yes	Yes	CD	Yes	Yes	No	CD	NA	Yes	Good	Good
Yokoo et al^38^	No	Yes	CD	CD	CD	Yes	NR	NA	Yes	Poor	Poor
Akin et al^39^	Yes	Yes	NA	NA	Yes	Yes	Yes	NA	Yes	Fair	Fair
Faqeeh et al^40^	Yes	Yes	NA	NA	Yes	Yes	Yes	NA	Yes	Fair	Fair
Jafari et al^41^	Yes	Yes	NA	NA	Yes	Yes	Yes	NA	Yes	Fair	Fair
Bashari et al^42^	Yes	Yes	NA	NA	Yes	Yes	Yes	NA	Yes	Fair	Fair
Beccara et al^43^	Yes	Yes	NA	NA	Yes	Yes	No	NA	Yes	Fair	Fair

CD, Cannot determine; NA, Not applicable; NIH, National Institutes of Health; NR, Not reported.

(Source: National Heart, Lung, and Blood Institute; National Institutes of Health; U.S. Department of Health and Human Services).

a The NIH Quality Assessment Tool for Case Series Studies^5^ includes nine questions: 1 = Was the study question or objective clearly stated? 2 = Was the study population clearly and fully described, including a case definition? 3 = Were the cases consecutive? 4 = Were the subjects comparable? 5 = Was the intervention clearly described? 6 = Were the outcome measures clearly defined, valid, reliable, and implemented consistently across all study participants? 7 = Was the length of follow-up adequate? 8 = Were the statistical methods well-described? 9 = Were the results well-described?

**Table 4.** Abdominal imaging findings in COVID-19 patients noted across various studies

First Author [Reference No.]	Imaging Changes in Bowel	Bowel dilatation/Fluid filled colon	Imaging changes in Pancreas	Solid Organ infarction	Hepatic Steatosis	Incidental lung base findings
Goldberg-Stein et al.^6^	+		+			
King et al.^7^						+
Bari Dane et al.^18^	+					
Shea et al.^8^	+			+		
Norsa et al.^9^	+					
Bhayana et al.^10^	+	+		+	+	
Medeiros et al.^19^					+	
Palomar-Lever et al.^11^					+	
Uchida et al.^12^					+	
Dane et al.^13^						+
Shiralkar et al.^14^	+					
Xiao et al.^15^						+
Sellevol et al.^16^						+
Liu et al.^17^^17^			+			
Kumar et al.^31^				+		
Sendi et al.^33^						+
Ignat et al.^27^	+					
Tay et al.^34^						+
Vu et al.^24^						+
Colino et al.^23^						+
Sattar et al.^32^	+	+				
Siegel et al.^35^						+
Gahide et al.^26^						+
Jaijakul et al.^36^	+					
Voutsinas et al.^37^	+					
Kim et al.^28^	+					
Poggiali et al.^25^	+					
Ahmed et al.^20^						+
Mazrouei et al.^29^			+			
Pessoa et al.^30^				+		
Azouz et al.^21^	+					
Akin et al.^39^				+		
Faqeeh et al.^40^				+		
Bashari et al.^42^	+					
Beccara et al.^43^	+					

### Imaging changes in bowel

15 studies reported bowel wall abnormalities on imaging among COVID-19 patients presenting with abdominal complaints.^6,8,10,14,18,21,22,25,27,28,32,36,37,43,44^ Among them, eight studies reported bowel wall thickening alone. Bhayana et al^10^ noted bowel wall thickening in 29% (12 out of 42 CT scans) of their abdominal CT scans in 40 patients, which involved the small bowel in 5 scans and large bowel and rectum in 7. They also found that bowel wall abnormalities were significantly associated with ICU admissions (Odds ratio: 15.56; *p* = 0.01). Goldberg-Stein et al^6^ also reported mural thickening in 15% (12 out of 80) patients with COVID-19. A few case reports also noted bowel wall thickening in patients with COVID-19.^25,36^ Sattar et al^32^ reported colonic findings on abdominal CT in three patients presenting with abdominal pain. Out of them, two showed wall thickening of large bowel, which involved the entire colon and rectum in one patient. Similar cases of colonic wall thickening on abdominal CT in patients with abdominal symptoms were also reported by Kim et al^28^ and Voutsinas et al^37^.

A total of six studies^8,10,21,27,41,44^ described imaging evidence of ischemic bowel changes. Besides bowel wall thickening, other findings reported were non-enhancing bowel wall, bowel wall pneumatosis, mesenteric venous gas, portal venous gas and mild fluid in the peritoneal cavity. Norsa et al^44^ in their retrospective cohort study, involving SARS-CoV-2 positive patients, noted six patients with features of intestinal ischemia in abdominal contrast-enhanced CT scan with two of these patients showing thromboembolic filling defects in IVC (Inferior vena cava) and superior mesenteric vein (SMV). The IVC thrombosis was probably related to the hypercoagulable state and not responsible for bowel ischemia. However, the prevalence of iliofemoral venous thrombosis or pulmonary emboli was not mentioned in their study. Hence, its relevance remains questionable. Shea et al^8^ noted CT features of bowel ischemia in four patients with COVID-19. Bhayana et al^10^, noted ischemic bowel changes in four patients. Few other authors also reported similar cases where abdominal pain led to cross-sectional imaging diagnosis of bowel ischemia.^10,21,22,27^ Ignat et al^27^ reported portal vein (PV) and SMV thrombosis with subsequent development of segmental ischemia of the small bowel.

### Bowel dilatation/fluid-filled colon

In the patients presenting with diarrhea, fluid-filled colon was noted on CT scan. Bhayana et al^10^ noted this finding in 18/42 (43%) of abdominal CT scans and it was more common in patients who were in the intensive care unit. Sattar et al^32^ reported colonic ileus in a patient presenting with constipation and abdominal pain.

### Imaging changes in pancreas

Four studies^6,14,29,45^ reported pancreatic abnormalities in patients with confirmed COVID-19. Goldberg-Stein et al^6^ noted pancreatic ductal dilatation in three patients with COVID-19. Liu et al^17^, in their retrospective observational study, noted imaging findings in the form of focal enlargement of the pancreas or dilatation of the main pancreatic duct, without any evidence of necrosis, in five patients with severe COVID-19. Mazrouei et al^29^ in their report of a 24-year-old male with COVID-19 and acute pancreatitis, showed mild edema of distal pancreas with peripancreatic fluid on abdominal CT.

### Solid organ infarction

Nine studies reported imaging findings of solid organ infarction (spleen, kidney, adrenal, liver) in patients with COVID-19.^6,8,10,18,22,30,31,39,40^ Isolated splenic infarct was noted in 10 patients, isolated renal infarct in 11 patients and liver infarct in 1 patient. One patient had both splenic and renal infarcts and one had bilateral adrenal infarcts. Goldberg-Stein et al^6^ in their RCS of COVID-19 patients with abdominal imaging, noted splenic and renal infarcts in four patients each. Faqeeh et al^40^ reported hypoperfusion changes in bilateral kidneys on abdominal CT associated with SARS-CoV-2 infection in a 19-year-old boy.

### Hepatic steatosis (HS)

Four studies,^10–12,19^ noted the presence of HS among COVID-19 positive patients. Medeiros et al^19^ noted a significantly higher prevalence of HS among patients with COVID-19 compared to non-COVID controls (31.9% *vs* 7.1%; *p* value < 0.001). Palomar-Lever et al^11^ in their retrospective study involving 213 COVID-19 patients, noted HS to be independently associated with severity of COVID-19 pneumonia. Uchida et al^12^ in their retrospective study involving 35 patients with mild-moderate COVID-19, noted reduced hepatic CT attenuation values to correlate with COVID-19 disease severity.^10^

### Incidental COVID detection: lung base findings of COVID-19 on abdominal CT in patients with predominant GI symptoms

A total of 18 studies (5 RCS, 7 case reports and 6 case series), reported basal lung findings on abdominal CT scans performed for predominant abdominal complaints.^6,7,10,13,15,16,20,23,24,26,28,31,33–35,37,38,42^ All the patients showed findings suggestive of COVID-19 in the basal lung sections included in the abdominal scans. These patients did not have any significant upper and lower respiratory symptoms, making the imaging findings in basal lung segments critical in raising a suspicion of COVID-19. The nature of incidental basal lung findings was reported in 118 patients. The most common finding was ground glass opacities, seen in 107 patients (90.7%) and in most of these patients (*n* = 76; 64.4%), the distribution of the opacities was peripheral and subpleural. The reverse halo sign and mild pleural effusion were noted in only three patients. The laterality of lung involvement was reported in 101 patients and among them, 92 (91.1%) had bilateral distribution of the lung base findings.

## Discussion

To our knowledge, this is the first review systematically scoping the various luminal and hepatopancreatobiliary imaging findings in patients with COVID-19. In our review, among studies reporting positive abdominal imaging findings in patients with COVID-19, majority reported imaging abnormalities of the GI tract, of which bowel wall thickening was the most frequent. It has been shown that intestinal abnormalities were associated with significantly worse outcomes in the form of requirement of ICU admissions. Imaging evidence of thromboembolism is being increasingly noted in patients with COVID-19. Imaging findings related to mesenteric ischemia and solid organ infarcts should be actively sought in COVID-19 patients presenting with pain abdomen. The higher occurrence of HS in patients with COVID-19 and its correlation with the severity of the disease has been noted in recent studies.^11,12,19^ However, further studies are needed to confirm or refute this association. The imaging features of involvement of other solid organs (liver, GB, pancreas) in COVID-19, is increasingly being recognized. . Lung base findings maybe incidentally noted on abdominopelvic CT of unsuspected patients, presenting with predominant abdominal complaints. Ground-glass opacities in the lung bases, usually multilobar and peripheral in distribution, are often the only pertinent findings observed.^13^ Hamilton et al^46^, in their retrospective study, noted a low overall diagnostic yield by performing an additional whole chest CT subsequent to the abdominal CT performed as part of their abdominal pain protocol. They noted that this lack of definitive benefit at the cost of increased radiation exposure makes it unjustified to add an extra chest CT scan. Similarly, Brennan et al^47^ did not find the benefit of addition of chest imaging to abdominal cross-sectional imaging, for the identification of patients with COVID-19.

The SARS-CoV-2 binds to the endothelial cells of the GI tract via ACE-2 receptors causing cytokine and chemokine release, responsible for acute intestinal inflammation.^48^ Findings on cross-sectional imaging of the affected patients support this inflammation theory with direct (ileocolitis) or indirect features (mesenteric lymphadenitis). The presence of bowel wall thickening should also merit consideration of intestinal ischemia due to vascular thrombosis. Hence, the two possibilities - presence of ACE-2 receptors in GI tract and procoagulant state - may be responsible for the bowel involvement in COVID 19. However, till this is completely proven, attribution of bowel wall thickening to COVID-19 should be a diagnosis of exclusion. Further, colonic dilatation and fluid in its lumen may also be due to direct or indirect effects of the virus. Since colonoscopy is an aerosol generating procedure, this procedure in patients with diarrhea or CT findings of colonic thickening, fluid or dilatation should prompt adequate precautions.

The association of COVID-19 with vascular thrombosis is increasingly being recognized and mesenteric vasculature remains no exception. Both small and large vessel thrombosis has been reported in patients with COVID-19.^49^ The resulting bowel ischemia has several direct and indirect radiological findings. Bhayana et al,^10^ in their preliminary observation, noted small bowel ischemic changes in 20% of the abdominal CT scans performed on patients with COVID-19 admitted to ICU. Several other reports have noted ischemic bowel changes on abdominal CT, among patients with COVID-19 presenting with abdominal pain. Although screening for pulmonary embolisms and deep vein thrombosis has been recommended in the clinical setting of COVID-19, little attention has been paid to thrombosis of the splanchnic venous system. Published reports have mentioned SMV and PV thrombosis as a result of the hypercoagulable state due COVID-19 *per se*. Visceral organ infarction should be considered in the differential diagnosis among COVID-19 patients presenting as unexplained abdominal pain. Clinicians need to be aware of the thrombotic manifestations of COVID-19 and radiologists should monitor patients for thrombosis to facilitate early diagnosis. Literature suggests that COVID-19 provokes arterial and venous thrombosis, although the mechanism remains largely unknown. Whether the thrombotic complications are a direct effect of SARS-CoV-2 or a consequence of ensuing cytokine storm remains contentious.

Pancreatic injury in patients with COVID-19 could be due to a direct cytopathic effect mediated by local SARS-CoV-2 replication or a result of the systemic response induced by SARS-CoV-2 infection.^17^ This could be inferred from the fact that, in one study, elevated pancreatic enzymes were noted in 13 patients, but the imaging changes suggestive of acute pancreatitis were seen in only 5.^17^ Although the imaging alterations suggested that pancreatitis was not severe, the problem should not be ignored, especially in patients with severe COVID-19. Further high-quality, prospective and well-reported research and larger series are warranted to evaluate whether this subset of patients have clinical pancreatitis as a presenting or concomitant disease entity. Studies are also needed to evaluate the management for this group and whether sequelae such as chronic pancreatitis may develop in the absence of early management.

Several studies have shown a higher occurrence of HS in patients with COVID-19 and have also reported a prognostic value.^6,50,51^ Presence of HS correlates with visceral adiposity, underlying metabolic disease, as well as overt and chronic inflammation.^52^ No causal association between HS and COVID-19 has been shown till date. Whether HS increases the predisposition for SARS-CoV-2 infection or is a manifestation of liver involvement, remains a conundrum.^53^ This needs to be conjectured from larger prospective studies.

### Study limitations

There are several limitations of this systematic review. Though our review gives an idea about the spectrum of abdominal imaging abnormalities seen in COVID-19 patients, no conclusion can be made regarding the incidence of these findings, as most of the reported literature consisted of case series and case reports. Although we aimed to correlate the abdominal imaging findings with the severity of COVID-19, this was not possible due to the immaturity of the available literature. Due to the novelty of SARS-CoV-2 and the brief timeframe since the start of the pandemic, the validity of evidence is limited. Case reports and case series form the most of the currently available literature. Nevertheless, given the paucity of high-quality evidence, inferences from such reports can guide decision- making and further research.^54^ Due to the relative paucity of literature on this topic, we included varied study designs in our review. The heterogeneity in the reporting of radiological findings may partly be attributed to extraction of data from clinical reports in few studies and re-reviewing of CT images in others. It is possible that certain findings (mural thickening, fluid-filled colon) may be relatively under reported in the clinical setting as compared with the research setting. Secondly, as studies reporting HS in patients with COVID-19 are all retrospective, causality cannot be determined, and the reported results may be considered as mere statistical associations. With our limited understanding of the disease pathophysiology, it is imperative to evaluate manifestations of all the systems which may be attributed to this disease. We need to discuss whether the abdominal manifestations in patients with COVID-19 or even silent abdominal imaging findings are due to unrelated concomitant pathological process or due to COVID-19 or a result of COVID-19 modifying the pathophysiology of a concomitant disease. However, before we can attempt to answer this question, we need to have a holistic understanding of both the abdominal manifestations and imaging findings in the COVID-19 patients. In this review, we noted that most studies reported findings on CT imaging. This might be reflective of the fact that it is the most widely available, reliable and relevant imaging modality. Ultrasound is less reliable and is influenced by COVID-19 safety measures. Despite the above limitations, this review systematically scopes and provides a spectrum of abdominal imaging findings in patients with COVID-19. We will continue to monitor the literature, and this review shall be updated as and when new evidence emerges.

## Conclusion

In conclusion, in this systematic review, we found a multitude of abdominal imaging findings among COVID-19 patients with predominant abdominal complaints. This review summarizes the abdominal imaging findings that have been described so far in COVID-19 patients and provides a platform for further research.
