# Supplementary material for: The eyes see what the mind seeks: a systematic review of abdominal imaging findings in patients with COVID-19
Source: Br J Radiol. 2021 Jul 14;94(1124):20201220. doi: 10.1259/bjr.20201220 (PMC8523189; doi:10.1259/bjr.20201220)
Supplement: Supplementary Material 1. [file bjr.20201220.suppl-01.docx]

**Supplement S1**

*PubMed search strategy.*

(“Coronavirus”[Mesh] OR “novel coronavirus”[Mesh] OR “2019-nCoV”[Mesh] OR “COVID-19”[Mesh]OR “nCoV”[Mesh]OR “SARS-2”[Mesh] OR Coronavirus*[tiab] OR novel coronavirus*[tiab] OR 2019-nCoV*[tiab] OR COVID-19*[tiab] OR coronavirus disease*[tiab] OR SARS-2*[tiab])

AND

(“Imaging”[Mesh] OR “Abdominal Imaging”[Mesh] OR “Computed tomography”[Mesh] OR “Abdominal computed tomography”[Mesh] OR “ultrasound”[Mesh] OR “abdominal ultrasound”[Mesh] OR “ Magnetic resonance ”[Mesh] OR Abdominal MRI*[Mesh] OR “Imaging”[tiab] OR “Abdominal Imaging”[tiab] OR “Computed tomography”[tiab] OR “Abdominal computed tomography”[tiab] OR “Abdominal scan”[tiab] OR “ultrasound”[tiab] OR “abdominal ultrasound”[tiab] OR “ Magnetic resonance ”[tiab] OR Abdominal MRI [tiab])

*Embase search strategy*

('coronavirus disease 2019'/exp OR 'coronavirus disease 2019') AND ('abdominal imaging'/exp OR 'abdominal imaging' OR 'abdominal CT' OR (abdominal AND ('CT'/exp OR CT)) OR 'abdominal ultrasound'/exp OR 'abdominal ultrasound' OR 'abdominal MRI vi' OR (abdominal AND ('scan'/exp OR scan)) AND vi))
