# Supplementary material for: The eyes see what the mind seeks: a systematic review of abdominal imaging findings in patients with COVID-19
Source: Br J Radiol. 2021 Jul 14;94(1124):20201220. doi: 10.1259/bjr.20201220 (PMC8523189; doi:10.1259/bjr.20201220)
Supplement: Supplementary Table 1. [file bjr.20201220.suppl-02.docx]

**Table S1: Characteristics of the included studies, reporting abdominal imaging findings in patients with COVID-19**

| **First Author [Reference No.]** | **Month of Publication** | **Location of Patient Cohort** | **Study Design** | **Number of patients with abdominal imaging** | **Diagnosis of COVID-19** | **Sex** | | **Number of Abdominal Imaging Performed** | | | **Remarks** |
| --- | --- | --- | --- | --- | --- | --- | --- | --- | --- | --- | --- |
|  |  |  |  |  |  | **Male** | **Female** | **US** | **CT** | **MRI** |  |
| **Goldberg-Stein et al.**^15^ | **August** | **USA** | **RCS** | **80** | **RT-PCR** | **33** | **47** | **-** | **80** | **-** | **Enlists the abdomino-pelvic CT findings in COVID-19 patients with abdominal symptoms. They concluded that younger age, male gender, and lower hemoglobin levels were independent predictors of positive findings on abdominopelvic CT.** |
| **King et al.**^43^ | **June** | **USA** | **RCS** | **62** | **RT-PCR** | **30** | **32** | **-** | **62** | **-** | **Lung base CT findings in COVID-19 patients presenting with abdominal complaints. Lung base CT findings included ground glass opacities in 95.2% patients (n=59), which were multifocal in 95.2% (n=59) patients and bilateral in 93.6% (n=58) patients. Peripheral distribution was noted in 66.1% (n=41) patients. Other described features such as rounded morphology and crazy paving were noted in 45.2% (n=28) and 6.5% (n=4) patients, respectively. Pleural effusion was noted in 1.6% (n=1) patient.** |
| **Bari Dane et al.**^17^ | **July** | **USA** | **Retrospective Case-control** | **7** | **NR** | **NR** | **NR** | **1** | **6** | **-** | **Seven COVID-19 patients had thromboembolic findings on abdominal imaging in comparison to one out of 82 patients in the control group. The thromboembolic findings in the COVID-19 group included arterial thromboembolism in three patients, renal vein thrombosis in one patient, splenic infarct in three patients, and renal infarct in one patient on CT and portal thrombosis in one patient on abdominal doppler US. They concluded that COVID-19 may present with medium to large sized arterial and venous thrombi and the presence of thrombosis may prompt the radiologist to suspect COVID-19.** |
| **Shea et al.**^18^ | **July** | **USA** | **RCS** | **9** | **RT-PCR** | **NR** | **NR** | **-** | **9** | **-** | **Abdominal imaging findings included features of bowel ischemia or infarction in four patients, renal infarction four patients and hepatic infarction in one patient.** |
| **Norsa et al.**^54^ | **July** | **Italy** | **RCS** | **6** | **RT-PCR** | **4** | **2** | **-** | **6** | **-** | **Six patients showed imaging evidence of intestinal ischemia. Among them thromboembolic filling defects in inferior vena cava (IVC) and superior mesenteric vein (SMV) were noted in one patient. The mortality among patients with COVID-19 related intestinal ischemia was 1.7-fold higher than among the non-COVID cohort.** |
| **Bhayana et al.**^6^ | **May** | **USA** | **RCS** | **134** | **RT-PCR** | **NR** | **NR** | **37** | **42** | **1** | **Evaluated the 224 abdominal imaging studies performed on 134 COVID-19 positive patients, including abdominal radiograph (n=137), US (n=44), CT (n=42) and MRI(n=1).** **Among the CT findings described, bowel wall abnormalities were the most common (13 out of 42 patients, 31%) with majority being bowel wall thickening (12 out of 42 patients, 29%). The other reported CT findings included fluid-filled colon in 18 out of 42 patients (43%). Solid-organ infarcts were noted in 2 out 42 patients. The most common US finding was distension of gallbladder with sludge suggesting cholestasis, which was noted in 20 out of 37 patients.** |
| **Medeiros et al.**^29^ | **July** | **Brazil** | **Retrospective Case-control** | **204** | **RT-PCR** | **94** | **110** | **-** | **204** | **-** | **Significantly higher prevalence of hepatic steatosis (HS) in the confirmed COVID-19 patients as compared to controls matched for age and sex.** |
| **Palomar-Lever et al.**^30^ | **July** | **USA** | **RCS** | **213** | **RT-PCR** | **131** | **82** | **-** | **213** | **-** | **Concluded that the group with severe COVID-19 (CT severity score >20) had higher association with male gender, obesity, hypertension and HS. On further analysis HS was found to be associated with severe COVID-19 independent of obesity. The pathophysiology remains unexplained, however the increased interleukin-6 levels in NAFLD patients might have an additive/synergistic role in promoting greater severity of COVID-19.** |
| **Uchida et al.**^31^ | **August** | **Japan** | **RCS** | **35** | **RT-PCR** | **22** | **13** | **-** | **35** | **-** | **Hepatic CT attenuation values as well as the L/S ratios were lower (suggesting hepatic steatosis) in the patients with severe COVID-19.** |
| **Dane et al.**^32^ | **April** | **USA** | **RCS** | **17** | **RT-PCR** | **NR** | **NR** | **-** | **17** | **-** | **Seventeen patients underwent PCR testing and all tested positive for COVID-19. Among these COVID-19 positive patients the lung base findings included ground glass opacities in 13 (76%) patients, which were multilobar in nine (53%) patients and peripheral in eight (47%) patients. Consolidations were noted in four (23.5%) patients. The study concluded that typical lung base findings can suggest a diagnosis of COVID-19 in patients with non-specific gastrointestinal (GI) symptoms.** |
| **Shiralkar et al.**^20^ | **Aug** | **USA** | **RCS** | **10** | **RT-PCR** | **7** | **3** | **1** | **12** | **1** | **Lung base findings typical for COVID-19 were noted in nine out of ten patients. GI tract imaging abnormalities were noted in 25% of abdominal CT scans and imaging features of acute interstitial pancreatitis was noted in one patient.** |
| **Xiao et al.**^41^ | **May** | **USA** | **RCS** | **6** | **RT-PCR** | **2** | **4** | **-** | **6** | **-** | **Lung base findings suggestive of COVID-19 on abdominal CT scans of six patients who presented with predominant abdominal complaints** |
| **Sellevol et al.**^37^ | **April** | **USA** | **RCS** | **1** | **RT-PCR** | **-** | **1** | **-** | **1** | **-** | **Patients with imaging findings suspicious for COVID-19 warrant prompt RT-PCR testing even in cases with low clinical suspicion.** |
| **Furong et al.**^24^ | **March** | **China** | **RCS** | **5** | **NR** | **NR** | **NR** | **-** | **5** | **-** | **They collected clinical data of mild and severe cases of COVID-19. In the severe COVID-19 group, 11 out of 67 patients (16.41%) had increased amylase and lipase and 5 out of 67 patients (7.46%) had imaging findings to suggest pancreatitis, including focal enlargement of the pancreas and pancreatic duct dilatation. In the mild COVID-19 group, only one (1.85 %) patient had increased amylase and lipase with no imaging changes. This study proposed a possibility of pancreatic injury in patients with severe COVID-19, considering the high expression of ACE-2 on the pancreatic islet and ductal cells.** |
| **Kumar et al.**^25^ | **May** | **UK** | **CR** | **1** | **RT-PCR** | **-** | **1** | **-** | **1** | **-** | **Bilateral adrenal infarction in a patient with COVID-19** |
| **Sendi et al**^39^ | **May** | **Saudi Arabia** | **CR** | **1** | **RT-PCR** | **1** | **-** | **-** | **1** | **-** | **Typical COVID-19 findings can be suggested on lung bases at abdominal CT.** |
| **Ignat et al.**^11^ | **April** | **France** | **CS** | **3** | **RT-PCR** | **2** | **1** | **-** | **3** | **-** | **Clinical and CT features of 3 patients**  **presenting with an acute abdomen induced by SARS-CoV-2 infection** |
| **Tay et al**^38^ | **April** | **UK** | **CR** | **1** | **RT-PCR** | **1** | **-** | **-** | **1** | **-** | **CT abdomen with contrast showed**  **bibasal lung consolidation in a case of COVID-19** |
| **Vu et al.**^34^ | **April** | **USA** | **CS** | **2** | **RT-PCR** | **NR** | **NR** | **-** | **2** | **-** | **Incidental detection of COVID-19 in patients with lung base findings on abdominal CT** |
| **Colino et al.**^42^ | **May** | **Spain** | **CR** | **1** | **RT-PCR** | **1** | **-** | **-** | **1** | **-** | **Suspected Acute Abdomen as an Extrapulmonary Manifestation of Covid-19 Infection** |
| **Sattar et al.**^7^ | **June** | **USA** | **CS** | **3** | **RT-PCR** | **3** | **-** | **-** | **3** | **-** | **COVID-19 with imaging manifestations of colitis** |
| **Siegel et al.**^33^ | **April** | **USA** | **CS** | **1** | **RT-PCR** | **1** | **-** | **-** | **1** | **-** | **Incidental detection of COVID-19 with lung base findings in patients with predominant gastrointestinal symptoms** |
| **Gahide et al.**^36^ | **May** | **Canada** | **CS** | **3** | **RT-PCR** | **1** | **2** | **-** | **3** | **-** | **COVID-19 patients presenting with afebrile acute abdominal pain** |
| **Jaijakul et al.**^12^ | **June** | **USA** | **CR** | **1** | **RT-PCR** | **1** | **-** | **-** | **1** | **-** | **Colitis as a Sole Presentation of SARS-CoV-2 Infection** |
| **Voutsinas et al.**^9^ | **May** | **USA** | **CS** | **4** | **RT-PCR** | **2** | **2** | **-** | **4** | **-** | **Incidental CT findings in the lungs in COVID-19 patients presenting with abdominal pain** |
| **Kim et al.**^8^ | **March** | **USA** | **CR** | **1** | **RT-PCR** | **1** | **-** | **-** | **1** | **-** | **Abdominal and testicular pain as an atypical manifestation of COVID-19** |
| **Poggiali et al.**^14^ | **March** | **Italy** | **CS** | **1** | **RT-PCR** | **-** | **1** | **1** | **1** | **-** | **COVID-19 patients presenting with gastrointestinal symptoms** |
| **Ahmed et al.**^35^ | **June** | **Qatar** | **CS** | **2** | **RT-PCR** | **1** | **1** | **-** | **2** | **-** | **Imaging may help in the diagnostic dilemma caused by atypical presentations of COVID-19** |
| **Mazrouei et al.**^22^ | **June** | **UAE** | **CR** | **1** | **RT-PCR** | **1** | **-** | **-** | **1** | **-** | **Possibility of a rare and direct causal relation between COVID-19 infection and acute pancreatitis** |
| **Pessoa et al.**^26^ | **June** | **Brazil** | **CS** | **2** | **RT-PCR** | **1** | **1** | **1** | **2** | **-** | **Splenic infarction as a thrombotic complication of COVID-19.** |
| **Azouz et al.**^13^ | **May** | **France** | **CR** | **1** | **RT-PCR** | **NR** | **NR** | **-** | **1** | **-** | **Hypercoagulability in COVID-19 causing systemic arterial thrombosis.** |
| **Akin et al.**^28^ | **July** | **Turkey** | **CR** | **1** | **RT-PCR** | **1** | **0** | **-** | **1** | **-** | **Renal infarct in a COVID-19 infected patient presenting with abdominal pain.** |
| **Faqeeh et al.**^27^ | **August** | **Saudi Arabia** | **CR** | **1** | **RT-PCR** | **1** | **0** | **-** | **1** | **0** | **COVID-19 infection affecting the kidneys can manifest radiologically as bilateral peripheral low perfusion changes.** |
| **Bashari et al.**^44^ | **July** | **USA** | **CR** | **1** | **RT-PCR** | **1** | **0** | **-** | **1** | **-** | **Epiploic appendigitis in a young male with incidentally detected COVID-19** |
| **Beccara et al.**^19^ | **April** | **Italy** | **CR** | **1** | **RT-PCR** | **1** | **0** | **-** | **1** | **-** | **SMA thrombosis with intestinal ischemia in a 52-year-old male with COVID-19** |

Abbreviations: RCS=Retrospective Cohort Study; PCS= Prospective Cohort Study; RT-PCR = reverse transcription polymerase chain reaction; PCR = polymerase chain reaction; CR = case report; CS = case series; US= Ultrasound; CT= Computerized Tomography; MRI= Magnetic Resonance Imaging; ACE= Angiotensin Converting Enzyme; COVID: Corona Virus Disease; SARS-CoV-2= Severe acute respiratory syndrome coronavirus-2; IVC**:** Inferior vena cava; SMV: Superior mesenteric vein; GI: Gastrointestinal; HS: Hepatic steatosis; NR = not reported
